# Sigma-1 and Sigma-2 receptors exhibit divergent genome-wide Co-expression architectures in human brain despite shared subcellular localization

**DOI:** 10.3389/fphar.2026.1830847

**Published:** 2026-06-04

**Authors:** Drake H. Harbert

**Affiliations:** Inner Architecture LLC, Canton, OH, United States

**Keywords:** co-expression architecture, mitochondria-associated membrane, neurodegeneration, sigma-1 receptor, sigma-2 receptor, subtype-selective pharmacology, TMEM97, weighted jaccard

## Abstract

The sigma-1 receptor (SIGMAR1) and sigma-2 receptor (TMEM97) are both enriched at the mitochondria-associated membrane (MAM) and have been pharmacologically co-classified for decades, yet their functional relationship at the transcriptomic level remains uncharacterized. We performed genome-wide co-expression analysis for both receptors across five brain regions from the GTEx v8 dataset (n = 209 in the primary region, 16,225 expressed genes) using Spearman correlations. Three Weighted Jaccard (WJ) formulations on continuous correlation vectors — (r+1)/2 shifted, unsigned |r|, and signed—all revealed that SIGMAR1 and TMEM97 share the majority of their global transcriptional architecture (WJ shifted = 0.964, unsigned = 0.907, signed = 0.906; all three rank-identical across 21 pairwise comparisons, ρ = 1.000), yet their top 5% co-expression networks overlap by only 10.0% (binary Jaccard = 0.100). Cosine similarity on raw vectors confirmed metric robustness (ρ = 0.856 with WJ, p = 7.5 × 10^−7^). Dissociation gap analysis across all 21 gene pairs showed the WJ-binary gap varies 2.1-fold (0.431–0.927), tracking known biological relatedness rather than reflecting a fixed methodological property. Gene Ontology analysis identified SIGMAR1-specific enrichment for mitochondrial translation and TCA cycle, and TMEM97-specific enrichment for ubiquitin-mediated proteolysis and neurodegeneration pathways. Multi-region replication confirmed the pattern across five brain regions, with the hippocampus showing tail-specific convergence. These findings are consistent with the hypothesis that dual sigma-1/sigma-2 ligands engage two functionally distinct co-expression programs within a shared cellular context, providing a transcriptomic rationale for subtype-selective pharmacological strategies.

## 1 Introduction

Sigma receptors were originally classified as a subtype of opioid receptor but are now recognized as a distinct class of intracellular chaperone and scaffolding proteins with diverse pharmacological profiles (Su et al., 2016; Hayashi, 2015; Schmidt and Kruse, 2019; Badawi et al., 2024; Shokr et al., 2025). Two subtypes have been identified: sigma-1 (SIGMAR1/S1R) and sigma-2 (S2R), molecularly identified in 2017 as TMEM97 (Alon et al., 2017). Both receptors are enriched at the mitochondria-associated membrane (MAM), the specialized ER subdomain forming physical contact sites with mitochondria (Alon et al., 2017; Hayashi and Su, 2007). Recent comprehensive reviews have detailed the pivotal role of sigma-1 receptors in neurological disorders, highlighting both neuroprotective and neuromodulatory functions across multiple disease contexts (Shokr et al., 2025).

SIGMAR1 is an ER chaperone that modulates calcium signaling, regulates the unfolded protein response, and interacts with ion channels and kinases (Hayashi and Su, 2007; Penke et al., 2018). Mutations cause juvenile amyotrophic lateral sclerosis and distal hereditary motor neuropathy (Al-Saif et al., 2011; Li et al., 2015), and SIGMAR1 ligands have shown therapeutic potential in neurodegeneration and depression (Niitsu et al., 2012; Hashimoto, 2013). Sigma-1-selective pharmacological modulation has demonstrated neuroprotective effects in seizure models, further supporting the therapeutic relevance of subtype-selective strategies (Badawi et al., 2024). SIGMAR1 is highly expressed in brain regions associated with cognition and stress response (Alonso et al., 2000; Mavlyutov et al., 2010). TMEM97 is implicated in cholesterol homeostasis through NPC1 and PGRMC1 interactions (Riad et al., 2018), amyloid-beta processing (Riad et al., 2020), and has been targeted in cancer imaging (Zeng et al., 2020) and Alzheimer’s disease therapeutics (Grundman et al., 2019).

Sigma receptors have additionally been implicated in addiction-related behavior. Both SIGMAR1 and TMEM97 are expressed in reward circuitry including the nucleus accumbens, and sigma receptor ligands modulate dopaminergic and opioidergic signaling relevant to substance use disorders (Fritz et al., 2011; Klawonn et al., 2017; Quadir et al., 2021; Sabino et al., 2017; Knowles et al., 2023). Understanding the transcriptomic divergence between the two subtypes is therefore pertinent not only to neurodegeneration and psychiatric pharmacology but also to addiction neuroscience, where subtype-selective engagement may produce distinct behavioral consequences depending on the regional co-expression context.

Despite shared MAM localization and pharmacological co-classification, the functional relationship between SIGMAR1 and TMEM97 remains unclear. They share no structural homology and bind overlapping but distinct ligand sets (Schmidt and Kruse, 2019). Many sigma ligands bind both subtypes, yet whether the two receptors coordinate their activities or serve independent roles has not been addressed at the transcriptomic level.

Standard co-expression analysis typically applies a threshold (e.g., top 5% of correlated genes) and compares resulting gene sets using binary overlap metrics. While informative, this approach discards information from the full correlation distribution and may conflate threshold-dependent artifacts with genuine architectural differences. Weighted Jaccard (WJ) similarity on continuous correlation vectors provides a threshold-free alternative that compares the complete co-expression architecture of two genes across the entire transcriptome (Van Dam et al., 2018; Langfelder and Horvath, 2008).

Here, we applied both approaches to SIGMAR1 and TMEM97 across five brain regions from GTEx v8 (GTEx Consortium, 2020). The combination of WJ on continuous vectors with binary Jaccard on thresholded networks reveals a dual-layer divergence pattern: the two receptors share their global transcriptional context but diverge specifically at their most tightly coordinated partners. This WJ-binary dissociation has implications for sigma receptor pharmacology.

## 2 Materials and methods

### 2.1 Data source and processing

Gene expression data (TPM) were obtained from GTEx v8 (dbGaP: phs000424. v8. p2) (GTEx Consortium, 2020). Brain-Frontal Cortex (BA9; n = 209) served as the primary region; BA9 was selected because it provides the largest GTEx brain sample size (n = 209), yielding the most stable genome-wide Spearman correlation estimates. Replication regions: Putamen, Hippocampus, Nucleus accumbens, and Anterior cingulate cortex (BA24). Genes with median TPM < 1.0 were excluded, retaining 16,225 genes. Expression values were log2 (TPM +1)-transformed.

### 2.2 Genome-wide Co-expression analysis

Spearman rank correlation coefficients were computed between each target gene and all 16,224 other expressed genes. Seven target genes were analyzed: SIGMAR1, TMEM97, and five contextual reference genes selected to represent ribosome quality control (PELO, LTN1, NEMF), translational stress response (EIF2S1), and ER stress (HSPA5). The resulting correlation vectors served as input for all similarity analyses.

### 2.3 Primary analysis: Weighted Jaccard on continuous vectors

Spearman correlation is the measurement method in this analysis—it quantifies the co-expression relationship between each target gene and all other expressed genes, producing a genome-wide correlation vector per target. Weighted Jaccard (WJ) is the interpretive framework that operates on those measurements, quantifying how much of the relational architecture is shared between two targets, where it diverges, and whether divergence concentrates globally or at functional tails.

WJ is the continuous generalization of binary Jaccard on the same measurement substrate. When the input vectors are thresholded to {0, 1}, the WJ formula reduces exactly to binary Jaccard. This means the difference between WJ and binary Jaccard (the dissociation gap) is a principled decomposition on a shared scale, quantifying where in the continuous correlation distribution the architectural divergence concentrates. This decomposition is not available when pairing geometrically different similarity measures: cosine similarity paired with binary Jaccard would yield two numbers on incommensurable scales with no mathematical bridge between them.

WJ provides set-theoretic interpretability that single-number similarity metrics do not. WJ = 0.964 reads as “96.4% of the architectural weight is shared between these two co-expression profiles” — used here as an effect-size descriptor of architectural overlap proportion, not as a claim that 96.4% of biological interactions are shared. Cosine similarity = 0.994 reads as “the vectors point in almost the same direction.” Spearman ρ = 0.905 reads as “the rank orderings are highly correlated.” All three statements are true; only WJ carries proportional meaning that decomposes into shared and unshared components, enabling the dual-layer analysis that is the central contribution of this paper. This interpretive architecture is domain-invariant: the same continuous-versus-thresholded decomposition localizes architectural divergence whether the fundamental units are turbofan engine sensors, gene co-expression profiles, or financial instrument correlations.

WJ was computed using three formulations to ensure robustness:

- WJ shifted (r+1)/2: Correlation coefficients shifted from [−1, 1] to [0, 1] via r_shifted = (r + 1)/2. The (r+1)/2 shift is a strictly monotone transformation, and WJ preserves rank under strictly monotone transformations because the min/max operations are order-preserving. Negatively correlated genes are mapped near 0, contributing minimally to the numerator and maximally to the denominator.
- WJ unsigned |r|: WJ computed on |r| vectors, treating positive and negative correlations symmetrically by magnitude. The absolute-value transformation is not strictly monotone (it maps both r = −0.9 and r = +0.9 to 0.9); convergence with the shifted formulation is an empirical property of the data indicating negligible sign-inversion contribution, rather than a theoretical guarantee.
- WJ signed: Positive and negative correlation components computed separately. Sign inversions contribute zero to the numerator and maximally to the denominator, scoring them as maximal divergence.

For all three formulations, WJ = sum (min (a_i, b_i))/sum (max (a_i, b_i)) across all genes i. Cosine similarity on raw (unshifted) correlation vectors was computed to validate the measurement layer independently of the WJ framework.

WGCNA module preservation (Zsummary; 17) is designed to test whether entire genome-scale co-expression modules are preserved across conditions, requiring hundreds to thousands of genes to define modules. Our analysis asks a fundamentally different question: whether two specific genes of pharmacological interest share co-expression neighborhoods. This pairwise gene-centric question cannot be addressed by WGCNA at the seven-gene panel scale; the two frameworks are complementary. Mutual information captures nonlinear dependencies beyond monotonic relationships but requires substantially larger sample sizes than the n = 176–246 available per brain region to produce stable genome-wide estimates; MI-based validation is identified as a future direction for larger datasets (Section 4.5).

Statistical significance was assessed via permutation testing (1,000 permutations, seed = 42). FDR correction (Benjamini-Hochberg) was applied across all 21 pairwise p-values.

### 2.4 Secondary analysis: Binary Jaccard on top 5% networks

The top 5% of correlated genes for each target defined its co-expression network. Network overlap was quantified using binary Jaccard (|A ∩ B|/|A ∪ B|) with Fisher’s exact test. FDR correction applied across all 21 comparisons.

### 2.5 Functional enrichment

Gene Ontology and pathway enrichment via gProfiler (Kolberg et al., 2023) against GO:BP, GO:MF, GO:CC, KEGG (Kanehisa et al., 2023), and Reactome (Gillespie et al., 2022) with g:SCS correction. Enrichment performed on SIGMAR1 top 5%, TMEM97 top 5%, SIGMAR1-unique, TMEM97-unique, and shared genes. Custom gene set enrichment assessed six curated sets using one-sided Fisher’s exact test.

### 2.6 Sensitivity analyses

Six sensitivity analyses were performed: (1) multi-region replication across five brain regions; (2) cell-type deconvolution with 53 markers across six cell types (Darmanis et al., 2015; Lake et al., 2018); (3) covariate adjustment for age and sex; (4) cosine similarity as metric validation; (5) three WJ formulations as architecture-choice validation; (6) dissociation gap analysis across all 21 pairs to test whether the WJ-binary dissociation tracks biological relatedness or is a fixed methodological property.

### 2.7 Software and reproducibility

All analyses in Python 3.13 (scipy, pandas, numpy, matplotlib). Seed = 42. Code: https://github.com/nwharbert8-ui/sigma-receptor-divergence. Archive: https://doi.org/10.5281/zenodo.19024710.

## 3 Results

### 3.1 SIGMAR1 and TMEM97 share global architecture but diverge at functional tails

All three WJ formulations revealed that SIGMAR1 and TMEM97 share the majority of their global co-expression architecture: WJ shifted = 0.964, WJ unsigned = 0.907, WJ signed = 0.906 (all permutation p < 0.001, FDR-corrected; Table 1). Binary Jaccard on their top 5% networks showed only 10.0% overlap (147 of 812 genes; Table 2). This WJ-binary dissociation indicates divergence concentrated at the extremes of the correlation distribution while the two receptors agree on their relationship to the bulk transcriptome.

**TABLE 1:** Weighted Jaccard similarity across all 21 pairwise comparisons of the seven-gene panel. Weighted Jaccard (WJ) computed on continuous Spearman correlation vectors against the full GTEx v8 BA9 transcriptome (n = 209 donors, 16,224 expressed gene partners). WJ values shown are from the unsigned |r| formulation; rank-identical results were obtained from the (r+1)/2-shifted and signed formulations (ρ = 1.000 across all 21 pairs). Permutation p-values from 1,000 gene-label shuffling permutations (seed = 42), with Benjamini–Hochberg FDR correction across all 21 comparisons. Pairs are ordered by ascending WJ (least similar architecture first); SIGMAR1–TMEM97 highlighted in bold.

Gene pair	WJ (unsigned)	Null mean	Null SD	z-score	Permutation p (FDR-adj.)
EIF2S1–HSPA5	0.9106	0.8274	0.0006	142.36	1.0000
PELO–HSPA5	0.9115	0.8302	0.0006	139.90	1.0000
LTN1–HSPA5	0.9182	0.8390	0.0006	134.77	1.0000
HSPA5–SIGMAR1	0.9192	0.8412	0.0006	130.58	1.0000
NEMF–HSPA5	0.9215	0.8442	0.0006	134.32	1.0000
TMEM97–HSPA5	0.9256	0.8380	0.0006	141.78	1.0000
NEMF–SIGMAR1	0.9316	0.8442	0.0007	128.70	1.0000
LTN1–SIGMAR1	0.9335	0.8405	0.0007	133.55	1.0000
NEMF–TMEM97	0.9439	0.8400	0.0007	148.84	1.0000
EIF2S1–SIGMAR1	0.9489	0.8346	0.0007	160.90	1.0000
PELO–NEMF	0.9490	0.8396	0.0007	158.89	1.0000
LTN1–TMEM97	0.9512	0.8363	0.0007	160.22	1.0000
EIF2S1–NEMF	0.9522	0.8361	0.0007	167.34	1.0000
PELO–LTN1	0.9571	0.8367	0.0007	171.49	1.0000
PELO–SIGMAR1	0.9595	0.8382	0.0007	172.36	1.0000
EIF2S1–LTN1	0.9621	0.8332	0.0007	181.52	1.0000
TMEM97–SIGMAR1	**0.9643**	**0.8388**	**0.0007**	**174.46**	**1.0000**
EIF2S1–TMEM97	0.9679	0.8302	0.0007	187.80	1.0000
PELO–TMEM97	0.9694	0.8338	0.0007	186.83	1.0000
LTN1–NEMF	0.9800	0.8422	0.0007	204.66	1.0000
EIF2S1–PELO	0.9839	0.8331	0.0007	204.16	1.0000

All 21 WJ values fall in the range 0.911 (EIF2S1–HSPA5) to 0.984 (EIF2S1–PELO). All permutation p-values reach the conservative null floor under the gene-label-shuffling permutation, reported as FDR-adjusted q-values.

**Table 2:** Binary Jaccard overlap of top-5%-correlated gene sets across all 21 pairwise comparisons. Binary Jaccard computed on the top 5% (812 genes) of correlated partners for each of the seven panel genes. “Shared” = number of genes appearing in both top-5% sets. Fisher's exact test odds ratio and p-value computed against the null of independent selection; Benjamini–Hochberg FDR correction applied across all 21 comparisons. Pairs are ordered by descending binary Jaccard (most overlap first); SIGMAR1–TMEM97 highlighted in bold.

Gene pair	Shared (of 812)	Binary Jaccard	Fisher OR	Fisher p (FDR-adj.)	Significant (q < 0.05)
EIF2S1–PELO	578	0.5526	160.22	< 1e-300	Yes
EIF2S1–TMEM97	542	0.5009	112.58	< 1e-300	Yes
PELO–TMEM97	514	0.4631	87.48	< 1e-300	Yes
LTN1–NEMF	496	0.4397	74.98	< 1e-300	Yes
EIF2S1–LTN1	325	0.2502	20.45	4.01e-225	Yes
PELO–HSPA5	264	0.1941	13.07	3.86e-151	Yes
EIF2S1–NEMF	255	0.1863	12.21	2.73e-141	Yes
PELO–SIGMAR1	242	0.1751	11.05	1.70e-127	Yes
PELO–LTN1	235	0.1692	10.47	2.55e-120	Yes
EIF2S1–HSPA5	233	0.1675	10.31	2.48e-118	Yes
LTN1–TMEM97	212	0.1501	8.72	7.72e-98	Yes
TMEM97–HSPA5	199	0.1396	7.84	6.14e-86	Yes
PELO–NEMF	197	0.1381	7.71	3.44e-84	Yes
TMEM97–SIGMAR1	**147**	**0.0995**	**4.90**	**1.37e-44**	**Yes**
NEMF–TMEM97	140	0.0943	4.57	7.04e-40	Yes
HSPA5–SIGMAR1	117	0.0776	3.56	6.94e-26	Yes
EIF2S1–SIGMAR1	113	0.0748	3.40	9.90e-24	Yes
NEMF–HSPA5	90	0.0587	2.54	7.51e-13	Yes
LTN1–HSPA5	87	0.0566	2.43	1.13e-11	Yes
NEMF–SIGMAR1	27	0.0169	0.64	1.00e+00	No
LTN1–SIGMAR1	11	0.0068	0.25	1.00e+00	No

Binary Jaccard for SIGMAR1–TMEM97 = 0.100 (147 of 812 genes shared) demonstrates tail-specific divergence despite high global WJ similarity (0.964; Table 1). All 21 Fisher's exact comparisons are FDR-significant, indicating that all pairs of top-5% sets overlap more than chance — but the extent of overlap varies more than five-fold across the panel.

**FIGURE 1:**
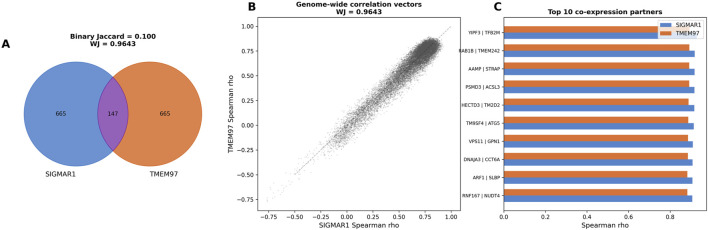
Genome-wide co-expression architecture comparison of SIGMAR1 and TMEM97 in human dorsolateral prefrontal cortex (GTEx BA9, n = 209). **(A)** Scatter plot of genome-wide Spearman correlation coefficients for SIGMAR1 (x-axis) versus TMEM97 (y-axis) across all 16,224 expressed genes; high alignment along the diagonal reflects the shared global transcriptional architecture (Weighted Jaccard = 0.964). **(B)** Top-5% co-expression network overlap visualized as a Venn diagram. Of 812 genes in the union, 665 are partner-set-exclusive (in one receptor’s top-5% but not the other’s) and 147 are shared (binary Jaccard = 0.100). **(C)** Top ten co-expression partners for SIGMAR1 (left) and TMEM97 (right), ranked by Spearman ρ. The top-10 lists are completely non-overlapping, illustrating tail-specific divergence despite globally similar architectures.

The three WJ formulations produced literal rank identity across all 21 pairwise comparisons (ρ = 1.000; verified by array-level index comparison, not rounding—every pair occupies the identical rank position across all three variants). For the shifted formulation, this is a theoretical guarantee: the (r+1)/2 shift is strictly monotone and WJ preserves rank under monotone transformations. For the unsigned formulation, rank identity with the shifted and signed variants is an empirical property confirming that sign inversions are negligible in this dataset (mean unsigned-vs-signed divergence = 0.002, max = 0.004). Cosine similarity on raw vectors correlated with all three WJ variants at ρ = 0.856 (p = 7.5 × 10^−7^), validating the measurement layer independently of the interpretive framework (Figure 1A; Supplementary Table S5).

Across all 21 pairs, WJ shifted ranged from 0.911 (EIF2S1-HSPA5) to 0.984 (EIF2S1-PELO). SIGMAR1-TMEM97 ranked fifth lowest. The most architecturally isolated gene was HSPA5, producing the six lowest WJ values, consistent with its distinct role as a master ER stress chaperone.

#### 3.1.1 Dissociation gap analysis confirms biological rather than methodological origin

Because WJ and binary Jaccard are the continuous and discrete versions of the same mathematical object, their difference (the dissociation gap) is a principled decomposition measuring how much architectural similarity is lost when moving from continuous to thresholded network representations. To test whether this loss is a fixed property of the method or tracks biological relatedness, we computed the dissociation gap for all 21 pairs using all three WJ formulations.

The gap varied from 0.431 (EIF2S1-PELO) to 0.927 (LTN1-SIGMAR1), a 2.1-fold range (Figure 1B; Supplementary Table S6). This range was consistent across formulations (shifted: 0.431–0.927; unsigned: 0.406–0.826; signed: 0.406–0.823). Functionally co-regulated pairs showed small gaps: LTN1-NEMF (0.540), PELO-TMEM97 (0.506), EIF2S1-PELO (0.431). Biologically divergent pairs showed large gaps: SIGMAR1-LTN1 (0.927), SIGMAR1-NEMF (0.915), EIF2S1-SIGMAR1 (0.874). SIGMAR1-TMEM97 (gap = 0.865, ratio = 9.7×) ranked fourth of 21. The top four gaps all involved SIGMAR1.

This ranking is predicted by biology, not methodology. The dissociation gap discriminates known co-regulated pairs from known divergent pairs across the full seven-gene panel.

### 3.2 Divergent tails encode distinct functional programs

**FIGURE 2:**
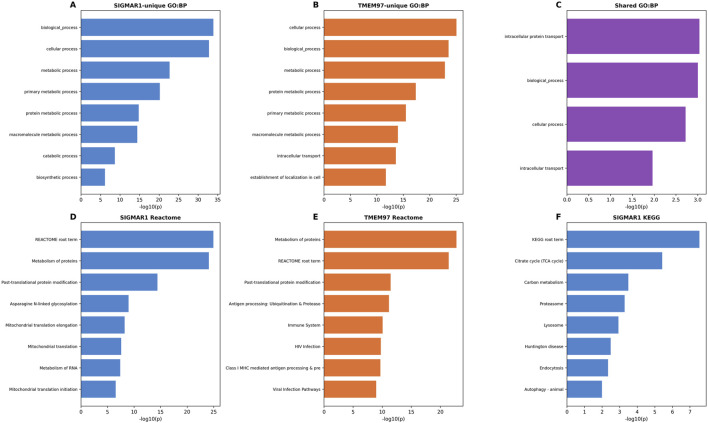
Functional enrichment of SIGMAR1- and TMEM97-specific co-expression partners. Six panels: **(A)** SIGMAR1-unique GO Biological Process terms; **(B)** TMEM97-unique GO:BP terms; **(C)** GO:BP terms shared between both top-5% networks; **(D)** SIGMAR1 Reactome pathway enrichment; **(E)** TMEM97 Reactome enrichment; **(F)** SIGMAR1 KEGG pathway enrichment. X-axis: −log^10^ (adjusted p-value) under g:SCS multiple-test correction. SIGMAR1 unique terms are dominated by mitochondrial translation and TCA cycle; TMEM97 unique terms are dominated by ubiquitin-mediated proteolysis and neurodegeneration pathways.

SIGMAR1’s unique top 5% genes: mitochondrial translation (Reactome), TCA cycle (KEGG). TMEM97’s unique set: ubiquitin-mediated proteolysis (KEGG), neurodegeneration pathways. Shared genes: TCA cycle, proteasome, carbon metabolism—the metabolic core of the MAM interface (Poston et al., 2013; Giacomello and Pellegrini, 2016) (Figure 2).

Top SIGMAR1 partners: YIPF3 (ρ = 0.928), RAB1B (0.918), AAMP (0.918), PSMD3 (0.917), HECTD3 (0.916). Top TMEM97 partners: TFB2M (0.893), TMEM242 (0.892), STRAP (0.892), ACSL3 (0.892), TM2D2 (0.889). Complete non-overlap of top-5 partners illustrates tail-specific divergence (Figure 1C).

### 3.3 Custom gene set enrichment

SIGMAR1: ribosome quality control (5.4-fold, p = 0.015; NPLOC4, PELO, VCP), replicated across all five regions. TMEM97: MAM-mitochondrial markers (6.1-fold, p = 3.10 × 10^−3^; RHOT1, VDAC1-3). Neither enriched for the other’s pathway. Vascular negative control: no enrichment.

### 3.4 Multi-region replication

**FIGURE 3:**
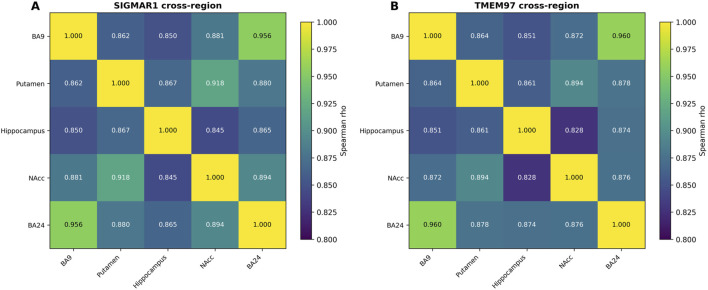
Multi-region replication of co-expression architecture. Heatmaps display pairwise Spearman correlation of the **(A)** SIGMAR1 and **(B)** TMEM97 genome-wide co-expression vectors across five GTEx brain regions: dorsolateral prefrontal cortex (BA9), putamen, hippocampus, nucleus accumbens (NAcc), and anterior cingulate cortex (BA24). Values range from ρ = 0.828 (least similar pair) to ρ = 1.000 (region against itself). Architectural similarity is consistently high (ρ > 0.83) across all five regions for both receptors, confirming the BA9-derived dual-layer dissociation generalizes across cortical and subcortical regions.

WJ stable across regions: BA9 = 0.964, Putamen = 0.979, Hippocampus = 0.975, NAcc = 0.968, BA24 = 0.967. Binary Jaccard varied: BA9 = 0.100, Putamen = 0.213, Hippocampus = 0.260, NAcc = 0.106, BA24 = 0.134. Hippocampal convergence is tail-specific: highest binary J (0.260) with only modest WJ increase (0.975) (Figure 3). This regional difference was identified *post hoc* and should be considered an exploratory observation; formal cross-region comparison would require independent replication cohorts.

### 3.5 Sensitivity analyses

Cell-type deconvolution preserved architecture (VCP retained in SIGMAR1 top 5%). Covariate adjustment: rank preservation ρ > 0.999. Pearson binary J = 0.112 vs. Spearman J = 0.100, WJ unchanged. Binary J at 3% and 10% thresholds: 0.065 and 0.230, confirming tail concentration. Three WJ formulations: identical rankings (ρ = 1.000). Cosine similarity: ρ = 0.856 with WJ. Multi-region WJ stability across five brain regions (0.964–0.979) provides empirical bounds on architectural consistency across independent sample subsets, addressing uncertainty in WJ point estimates. All sensitivity checks confirm robustness.

## 4 Discussion

This study introduces a dual-layer analysis of sigma receptor co-expression architecture. The central finding is a WJ-binary dissociation: SIGMAR1 and TMEM97 share their global architecture but diverge at their functional tails. Three WJ formulations and cosine similarity confirm this pattern is metric-robust. Dissociation gap analysis across 21 pairs demonstrates the gap tracks biological relatedness (2.1-fold range) rather than being a fixed methodological artifact.

### 4.1 Pharmacological implications

The WJ-binary dissociation has implications for sigma receptor pharmacology that warrant experimental investigation. Many sigma ligands bind both subtypes (Schmidt and Kruse, 2019), and the high WJ indicates the two receptors coexist in the same transcriptional environment. However, the low binary J indicates that a dual ligand would be predicted to simultaneously engage one target embedded in mitochondrial translation and TCA cycle machinery (SIGMAR1) and another in ubiquitin-proteasome and neurodegeneration pathways (TMEM97). This co-expression divergence is consistent with the hypothesis that subtype-selective ligands (Niitsu et al., 2012; Francardo et al., 2014; Grundman et al., 2019) may offer therapeutic precision that dual ligands cannot. Recent sigma-1-selective neuroprotection in seizure models (Badawi et al., 2024) and comprehensive reviews of sigma-1 neurological roles (Shokr et al., 2025) support this rationale.

These pharmacological implications are hypothesis-generating; experimental validation through perturbation studies or ligand-response transcriptomic profiling would be required to confirm that co-expression divergence translates to differential pharmacodynamic outcomes.

### 4.2 SIGMAR1 architectural isolation

SIGMAR1 occupies the most isolated transcriptional position in the panel, quantitatively confirmed by dissociation gap analysis: the four largest gaps (0.865–0.927) all involve SIGMAR1. TMEM97 shows high WJ with translational QC components (EIF2S1: 0.968, PELO: 0.969) while SIGMAR1 drops steeply (NEMF: 0.932, LTN1: 0.933). SIGMAR1 engages downstream proteostatic effectors (VCP, NPLOC4; RQC enrichment 5.4-fold) while being maximally divergent from the ribosome-associated detection arm, possibly reflecting physical separation of mitochondrial translation from cytoplasmic surveillance at the MAM (Greber and Ban, 2016; Brandman and Hegde, 2016; Joazeiro, 2019).

### 4.3 The hippocampal exception as tail-specific convergence

Binary J: 0.100 to 0.260 in hippocampus; WJ: 0.964 to 0.975 (1.1% shift). The hippocampus selectively merges functional tails while preserving global architecture. Dual ligands may therefore have different consequences in hippocampus-dependent disorders because tail-specific convergence partially collapses the subtype selectivity present in cortical regions.

### 4.4 HSPA5 as architectural validation

HSPA5 produced the six lowest WJ values across all 21 comparisons—an undesigned validation that the most biologically distinct gene ranks as most architecturally divergent.

### 4.5 Limitations

Co-expression reveals coordination, not causation (van Dam et al., 2018). Bulk RNA-seq lacks cell-type resolution; deconvolution showed moderate preservation but single-cell confirmation would strengthen findings (Bakken et al., 2021). GTEx captures steady-state post-mortem expression, not dynamic pharmacological regulation (GTEx Consortium, 2020). Sigma receptor function involves post-translational modifications not captured by transcriptomics (Hayashi and Su, 2007; Penke et al., 2018). The permutation null tests against random gene pairs via gene label shuffling. Because genes are highly co-regulated, this procedure disrupts co-expression structure and produces an artificially narrow null distribution, inflating permutation-based z-scores and p-values relative to a biologically appropriate null. WJ values are therefore reported as effect sizes; permutation p-values should be interpreted as conservative upper bounds on statistical significance rather than precise probability estimates. A pathway-matched null comparing against other multi-component pathway pairs would be more stringent and represents a future direction. GO and pathway enrichment analyses were performed with gProfiler’s g:SCS correction for multiple testing, which does not control for gene-length or expression-level biases; these factors may influence enrichment results and should be considered when interpreting GO findings. Mutual information-based similarity, which captures nonlinear dependencies beyond the monotonic relationships detected by Spearman correlation, was not computed due to the instability of MI estimation at the sample sizes available per brain region (n = 176–246); MI-based validation in larger datasets is an additional future direction.

## 5 Conclusion

SIGMAR1 and TMEM97 share global co-expression architecture (WJ shifted = 0.964, unsigned = 0.907, signed = 0.906) but diverge at their functional tails (binary J = 0.100). All three WJ formulations produce identical rankings (ρ = 1.000); cosine similarity confirms metric robustness. Dissociation gap analysis shows the WJ-binary gap varies 2.1-fold across 21 pairs, tracking biology not methodology. SIGMAR1 coordinates with mitochondrial translation; TMEM97 with ubiquitin-proteasome and neurodegeneration pathways. The dissociation is consistent with a transcriptomic rationale for subtype-selective pharmacological strategies (Badawi et al., 2024; Shokr et al., 2025) and identifies the hippocampus as a region where tail-specific convergence may alter dual-ligand consequences.

## Data Availability

Publicly available datasets were analyzed in this study. This data can be found here: GTEx v8 gene expression data (TPM) are available through the GTEx Portal (https://gtexportal.org/) under dbGaP accession phs000424.v8.p2. All analysis code is available at https://github.com/nwharbert8-ui/sigma-receptor-divergence-wj and archived at https://doi.org/10.5281/zenodo.19024710. Derived results (genome-wide correlation rankings, enrichment results, shared gene lists) are provided as Supplementary Data Sheets 1–4.

## Supplementary material

SUPPLEMENTARY DATA SHEET 1 gProfiler functional enrichment output (GO:BP, GO:MF, GO:CC, KEGG, Reactome) for SIGMAR1-top5%, TMEM97-top5%, SIGMAR1-unique, TMEM97-unique, and shared gene sets.

SUPPLEMENTARY DATA SHEET 2 Custom gene set enrichment results (RQC, MAM-mitochondrial markers, vascular negative controls) for SIGMAR1 and TMEM97 across the five brain regions.

SUPPLEMENTARY DATA SHEET 3 Shared genes between SIGMAR1 and TMEM97 top-5% sets (147 genes), with cross-region presence/absence matrix.

SUPPLEMENTARY DATA SHEET 4 Genome-wide co-expression rankings (Spearman ρ values) for all seven panel genes against all 16,224 expressed partners.
